# Impact of electrically formed interfacial layer and improved memory characteristics of IrO_x_/high-κ_x_/W structures containing AlO_x_, GdO_x_, HfO_x_, and TaO_x_ switching materials

**DOI:** 10.1186/1556-276X-8-379

**Published:** 2013-09-06

**Authors:** Amit Prakash, Siddheswar Maikap, Writam Banerjee, Debanjan Jana, Chao-Sung Lai

**Affiliations:** 1 Thin Film Nano Technology Laboratory, Department of Electronic Engineering, Chang Gung University, Tao-Yuan 333, Taiwan; 2 Biomedical Engineering Research Center, Department of Electronic Engineering, Chang Gung University, Tao-Yuan 333, Taiwan

**Keywords:** Resistive switching, W/TaO_x_, Ti nanolayer, Oxygen ion migration, Nanofilament

## Abstract

Improved switching characteristics were obtained from high-κ oxides AlO_x_, GdO_x_, HfO_x_, and TaO_x_ in IrO_x_/high-κ_x_/W structures because of a layer that formed at the IrO_x_/high-κ_x_ interface under external positive bias. The surface roughness and morphology of the bottom electrode in these devices were observed by atomic force microscopy. Device size was investigated using high-resolution transmission electron microscopy. More than 100 repeatable consecutive switching cycles were observed for positive-formatted memory devices compared with that of the negative-formatted devices (only five unstable cycles) because it contained an electrically formed interfacial layer that controlled ‘SET/RESET’ current overshoot. This phenomenon was independent of the switching material in the device. The electrically formed oxygen-rich interfacial layer at the IrO_x_/high-κ_x_ interface improved switching in both via-hole and cross-point structures. The switching mechanism was attributed to filamentary conduction and oxygen ion migration. Using the positive-formatted design approach, cross-point memory in an IrO_x_/AlO_x_/W structure was fabricated. This cross-point memory exhibited forming-free, uniform switching for >1,000 consecutive dc cycles with a small voltage/current operation of ±2 V/200 μA and high yield of >95% switchable with a large resistance ratio of >100. These properties make this cross-point memory particularly promising for high-density applications. Furthermore, this memory device also showed multilevel capability with a switching current as low as 10 μA and a RESET current of 137 μA, good pulse read endurance of each level (>10^5^ cycles), and data retention of >10^4^ s at a low current compliance of 50 μA at 85°C. Our improvement of the switching characteristics of this resistive memory device will aid in the design of memory stacks for practical applications.

## Background

Resistive random access memory (RRAM) with a simple metal-insulator-metal structure shows promising characteristics in terms of scalability, low power operation, and multilevel data storage capability and is suitable for next-generation memory applications [1-4]. RRAM devices with simple structure and easy fabrication process that are compatible with high-density 3D integration [5] will be needed in the future. Various oxide switching materials such as HfO_x_[6-9], TaO_x_[3,10-15], AlO_x_[16-19], GdO_x_[20], TiO_x_[21-23], NiO_x_[24,25], ZrO_x_[26-29], ZnO [30-32], SiO_x_[33], and GeO_x_[34-36] have been used in nanoscale RRAM applications. However, their nonuniform switching and poorly understood switching mechanisms are currently the bottlenecks for the design of nanoscale resistive switching memory. Generally, inert metal electrodes [4] and various interfacial methods are used to improve resistive switching memory characteristics. We previously reported polarity-dependent improved memory characteristics using IrO_x_ nanodots (NDs) in an IrO_x_/AlO_x_/IrO_x_-NDs/AlO_x_/W structure [16]. However, improved memory performance using different high-κ oxide switching materials such as AlO_x_, GdO_x_, HfO_x_, and TaO_x_ in IrO_x_/high-κ_x_/W structures has not been reported yet. Using different high-κ oxides in the same structure may reveal a unique way to design novel RRAM devices for practical applications. Electrical formation of an interfacial layer at the IrO_x_/high-κ_x_ interface is important to improve resistive switching memory characteristics. Using this approach, high-density memory could be achieved using an IrO_x_/AlO_x_/W cross-point structure, which we also report here.

In this study, we show that the electrically formed oxygen-rich interfacial layer at the IrO_x_/high-κ_x_ interface in an IrO_x_/high-κ_x_/W structure plays an important role in improving the resistive switching memory characteristics of the structure. The positive-formatted(PF) devices exhibited more switching cycles compared to the negative-formatted (NF) ones and do not depend on the switching material. When the pristine resistive memory device is formed using positive polarity bias on the TE, it is termed as PF, while the negative voltage-formed device is termed as an NF device. PF devices with similar switching behavior are obtained using different high-κ oxide films of AlO_x_, GdO_x_, HfO_x_, and TaO_x_. The switching mechanism is the formation/oxidation of oxygen vacancies in a conducting filament by controlling the migration of oxygen ions through the electrically formed interfacial layer. This unique phenomenon helps to design high-density cross-point memory using an IrO_x_/AlO_x_/W structure. This cross-point memory was forming-free, exhibiting 1,000 consecutive ‘dc’ cycles at a current compliance (CC) of <200 μA and a small operation voltage of ±2 V, highly uniform switching (yield >95%) with multilevel capability (at least four different levels of low resistance state (LRS)). The device can be switched even using a very small current of 10 μA, which makes it useful for low power applications. The surface morphology and roughness of the structure were observed by atomic force microscopy (AFM). The device size and interfaces of layers were investigated by transmission electron microscopy (TEM). These observations show that the improved performance of this device structure can be attributed to the electrically formed O-rich interfacial layer at the top electrode/filament interface. The devices have also shown good read endurance of >10^5^ cycles and data retention at 85°C under a low CC of 50 μA.

## Methods

**Figure 1:**
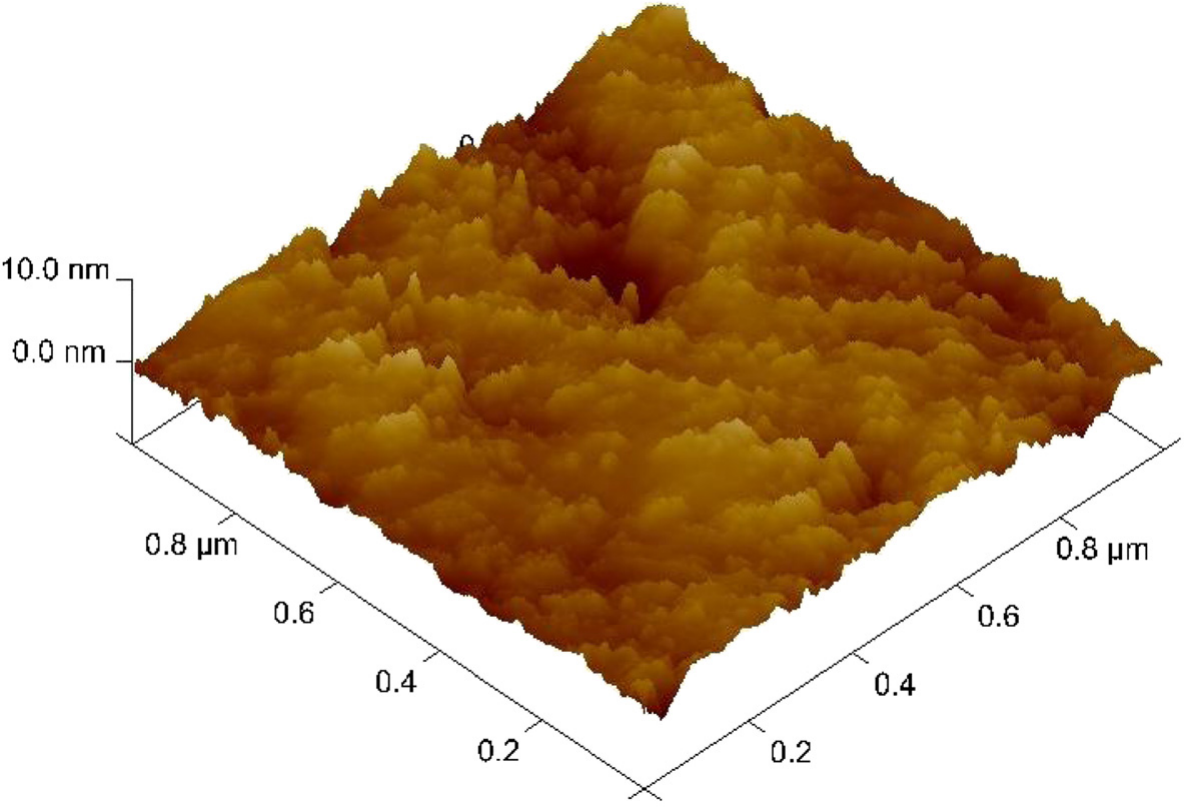
**AFM image of the W surface of an S1 device.** The RMS surface roughness is 1.18 nm.

**Figure 2:**
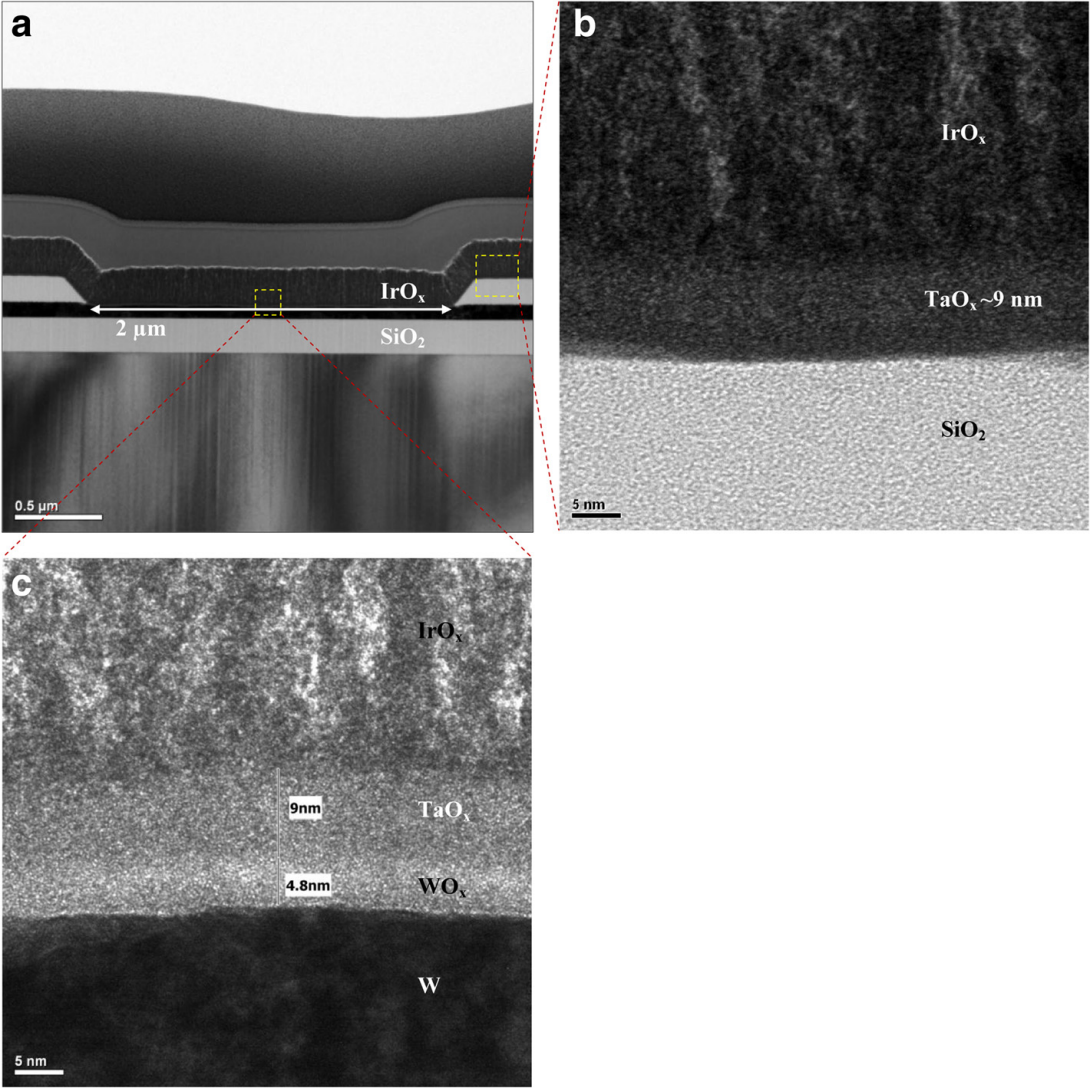
**TEM and HRTEM images of IrO**_**x**_**/TaO**_**x**_**/W stack with via-hole structure and size of 2 × 2 μm. (a)** TEM image. **(b)** HRTEM image outside of active region. The TaO_x_ film is approximately 9 nm thick and amorphous. **(c)** HRTEM image in the active region. A WO_x_ layer with a thickness of approximately 5 nm is formed inside the hole region.

Resistive switching memory devices using high-κ oxides AlO_x_, GdO_x_, HfO_x_, and TaO_x_ in a standard via-hole IrO_x_/high-κ_x_/W structure (Device: S1) were fabricated. A W layer with a thickness of approximately 100 nm as a bottom electrode (BE) was deposited on SiO_2_ (200 nm)/Si substrates. Figure 1 shows an AFM image taken in tapping mode using an Innova Scanning Probe Microscope system (Bruker, Madison, WI, USA) of a deposited W film surface. The average and root mean square (RMS) roughness of the surface were 0.91 and 1.18 nm, respectively. An SiO_2_ layer with a thickness of approximately 150 nm was then deposited at low temperature on each W BE. Photolithography and dry etching techniques were used to form holes of different sizes in the range of 0.4 to 8 μm in the structure. Then, AlO_x_ and HfO_x_ films were deposited by sputtering, and GdO_x_ and TaO_x_ films were deposited by electron beam evaporation. The thickness of each high-κ film was 10 to 15 nm. The top electrode (TE) of IrO_x_(approximately 200 nm thick) was deposited by reactive sputtering using a pure Ir target and O_2_ as the reactive gas. The final devices with a structure of IrO_x_/high-κ_x_/W were obtained after a lift-off process. The structure of the memory devices and thicknesses of all deposited layers were observed by TEM at an energy of 200 keV. Figure 2a shows a typical cross-sectional TEM image of an IrO_x_/TaO_x_/W resistive memory stack with a via-hole structure (S1). The typical device size was 2 × 2 μm. The high-resolution transmission electron microscopy (HRTEM) image taken inside the via-hole (Figure 2c) reveals the formation of two layers; one is TaO_x_ and the other one is WO_x_, which is formed by the surface oxidation of the W BE because of the *ex situ* fabrication process. To confirm the thickness of the deposited TaO_x_ layer, a HRTEM image was acquired from the area outside the via-hole, i.e., on the SiO_2_ (Figure 2b). The amorphous TaO_x_ layer was approximately 9nm thick, confirming that the thickness of the polycrystalline WO_x_ layer inside the via-hole was approximately 5 nm (Figure 2c). This kind of bilayer structure (high-κ/WO_x_) was observed in all of the fabricated resistive memory stacks investigated (TEM images not shown here).

**Figure 3:**
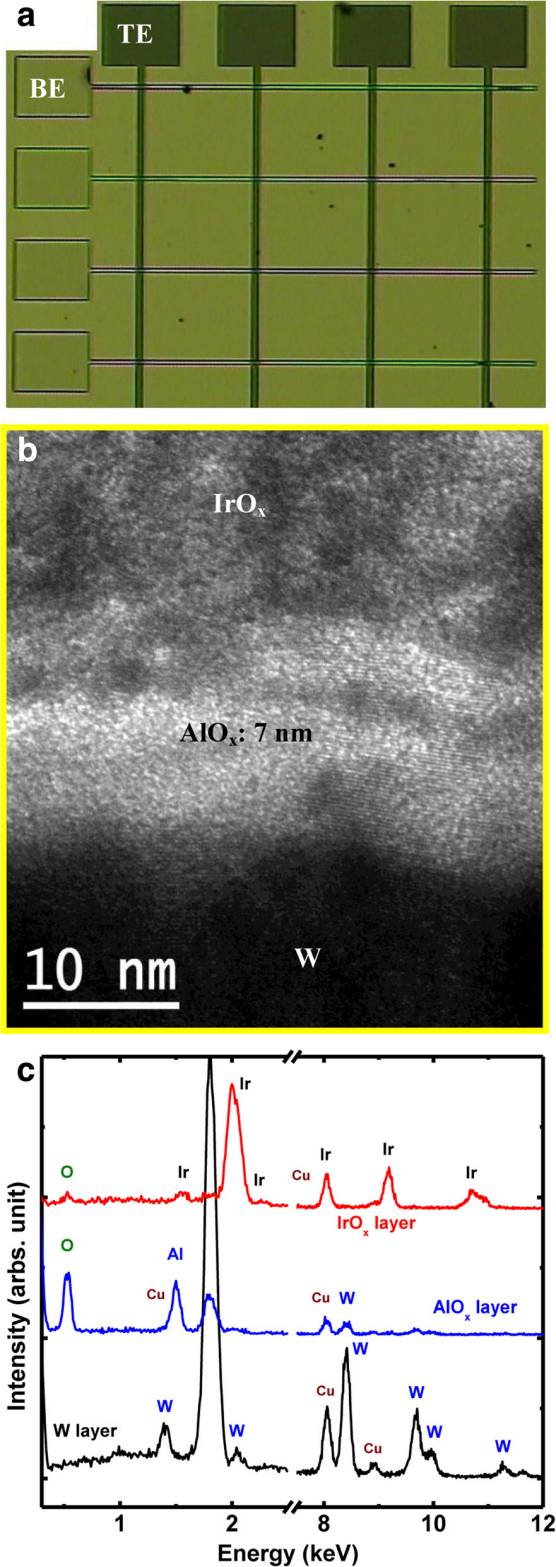
**Morphology and composition of an IrO**_**x**_**/AlO**_**x**_**/W cross-point structure. (a)** OM image. **(b)** Cross-sectional TEM image of the cross-point memory device. The thickness of AlO_x_ film is approximately 7 nm. **(c)** EDS obtained from TEM image **(b)**.

**Figure 4:**
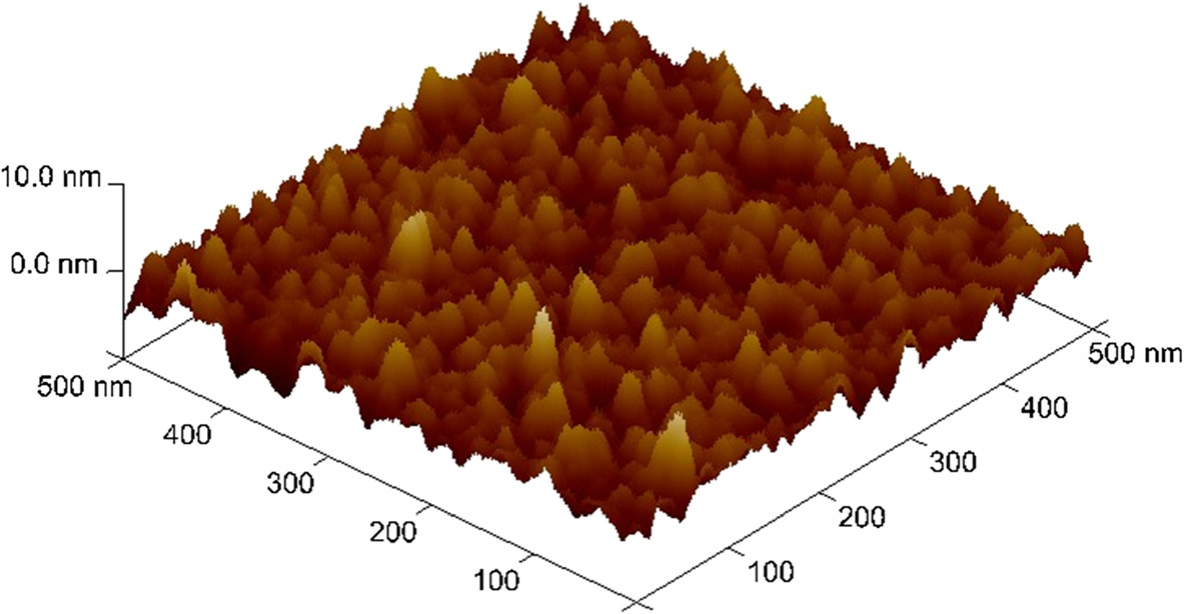
**AFM image of W surface of IrO**_**x**_**/AlO**_**x**_**/W cross-point device.** The RMS roughness is approximately 1.35 nm.

To obtain high-density memory, W films with a thickness approximately 100 nm were deposited on the SiO_2_ (200 nm)/Si substrates by sputtering to form IrO_x_/AlO_x_/W cross-point structures (Device: S2), which were patterned using photolithography and wet etching techniques to form W BE stripes. Cross-point memory with different sizes ranging from 4 × 4 to 50 × 50 μm was fabricated by another lithography step to pattern the TE stripes using a lift-off method. To obtain forming-free cross-point memory, the thickness of the AlO_x_ layer was 7 nm. Figure 3a shows a typical optical microscope (OM) image of a fabricated resistive memory device with an IrO_x_/AlO_x_/W cross-point structure (Device: S2) with a size of 4 × 4 μm. The AlO_x_ layer sandwiched between the IrO_x_ TE and W BE is clearly seen in a cross-sectional HRTEM image of this device (Figure 3b). The surface of the W BE is rough. The energy-dispersive X-ray spectra shown in Figure 3c confirm that the respective layers contain Ir, Al, O, and W. To further examine the roughness and surface morphology of the W BE, an AFM image of the W BE surface was obtained, as shown Figure 4. The average and RMS surface roughness of the W BE were 1.05 and 1.35 nm, respectively, which are higher than those of the W BE in the devices with via-hole structures (S1, as shown in Figure 1). This morphological difference is also found to be important to improve the resistive switching behavior of cross-point memory devices, which will be discussed later. However, we first designed the via-hole PF devices (S1) and then the cross-point structure (S2) to improve memory characteristics. A bias was applied to the TE, and the BE was electrically grounded in both of the structures.

## Results and discussion

**Figure 5:**
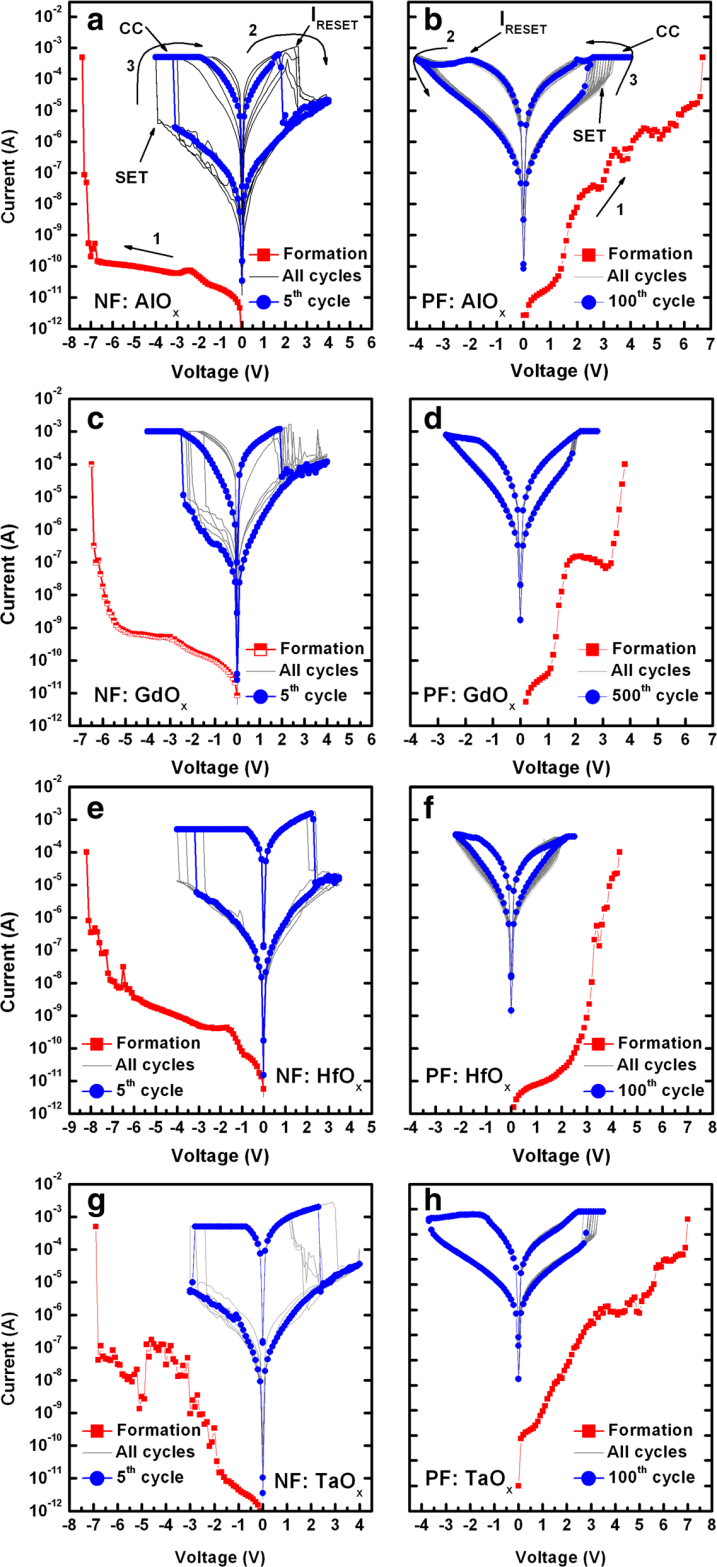
**Current–voltage (*I-V*) switching characteristics of devices with via-hole structure under negative (NF) and positive formation (PF). (a, c, e, and g)** Switching curves of NF devices containing AlO_x_, GdO_x_, HfO_x_, and TaO_x_ switching materials, respectively, in an IrO_x_/high-κ_x_/W structure. **(b, d, f, and h)** PF devices containing AlO_x_, GdO_x_, HfO_x_, and TaO_x_ switching materials, respectively, in an IrO_x_/high-κ_x_/W structure.

The current–voltage (*I-V*) properties of the NF and PF devices (S1) with bipolar resistive switching memory characteristics are shown in Figure 5. The sweeping voltage is shown by arrows 1 to 3. Figure 5a shows the typical *I-V* curves of the NF devices with an IrO_x_/AlO_x_/W structure. A high formation voltage of about <−7.0 V was required with very low leakage current. After formation, the first five consecutive switching cycles show large variations in low and high resistance states as well as SET/RESET voltages with higher maximum reset current (*I*_RESET_) than the set or CC. Similar behavior can be observed for all of the other resistive memory devices containing GdO_x_, HfO_x_, and TaO_x_ as switching materials (Figure 5c,e,g). Figure 5b shows typical consecutive *I-V* switching curves for 100 cycles together with the formation curve at a positive voltage obtained for the AlO_x_-based device with a via-hole structure. Remarkable improvement in the consecutive switching cycles with a tight distribution of LRS and high resistance state (HRS) and SET/RESET voltage was obtained, which is suitable for RRAM devices. Furthermore, *I*_RESET_ is not higher than that of the CC unlike the NF devices, which indicates that the PF devices are mainly electric field-dominated, and switching occurs near the interface. In contrast, electric field-induced thermal effects are also important in the case of the NF devices, and large variations in switching occur. The uncontrolled current flow through the filament in the NF device will enhance Joule heating as well as the abrupt breaking of the filament, and the RESET current curve is suddenly reduced. On the other hand, the RESET current in the PF device is changed slowly because of the series resistance which will control the current flow through the filament precisely. That is why the current changes slowly in the PF devices. It is interesting to note that the resistance of LRS of PF device is higher (approximately 10 kΩ) than that of the NF device (approximately 1 kΩ), and the controlling current through the series resistance of the PF devices will have also lower HRS than that of the NF devices. Therefore, the NF devices will have lower value of LRS and higher value of HRS, which results in the higher resistance ratio as compared to the PF devices. All of the other fabricated PF devices show a similar improvement in switching, as shown in Figure 5d,f,h. The leakage current is smaller in the negative-voltage region (Figure 5a) than in the positive-voltage region (Figure 5b) because of the higher barrier height for electron injection imposed by the higher work function of the IrO_x_ TE (*Φ*_IrO2_> 5 eV [37] and *Φ*_W_ of approximately 4.55 eV [38]) when a negative voltage is applied. It is important to note that all of the resistive memory devices show similar switching characteristics irrespective of the switching material. This suggests that in the electrode materials, their reactivity and top/bottom selection are very important for RRAM stacks, which allow their switching properties as well as device performance to be improved by controlling SET/RESET polarity. Therefore, this unique study using the switching materials AlO_x_, GdO_x_, HfO_x_, and TaO_x_ in an IrO_x_/high-κ_x_/W structure provides clues for improving the design of nanoscale high-performance nonvolatile memory.

**Figure 6:**
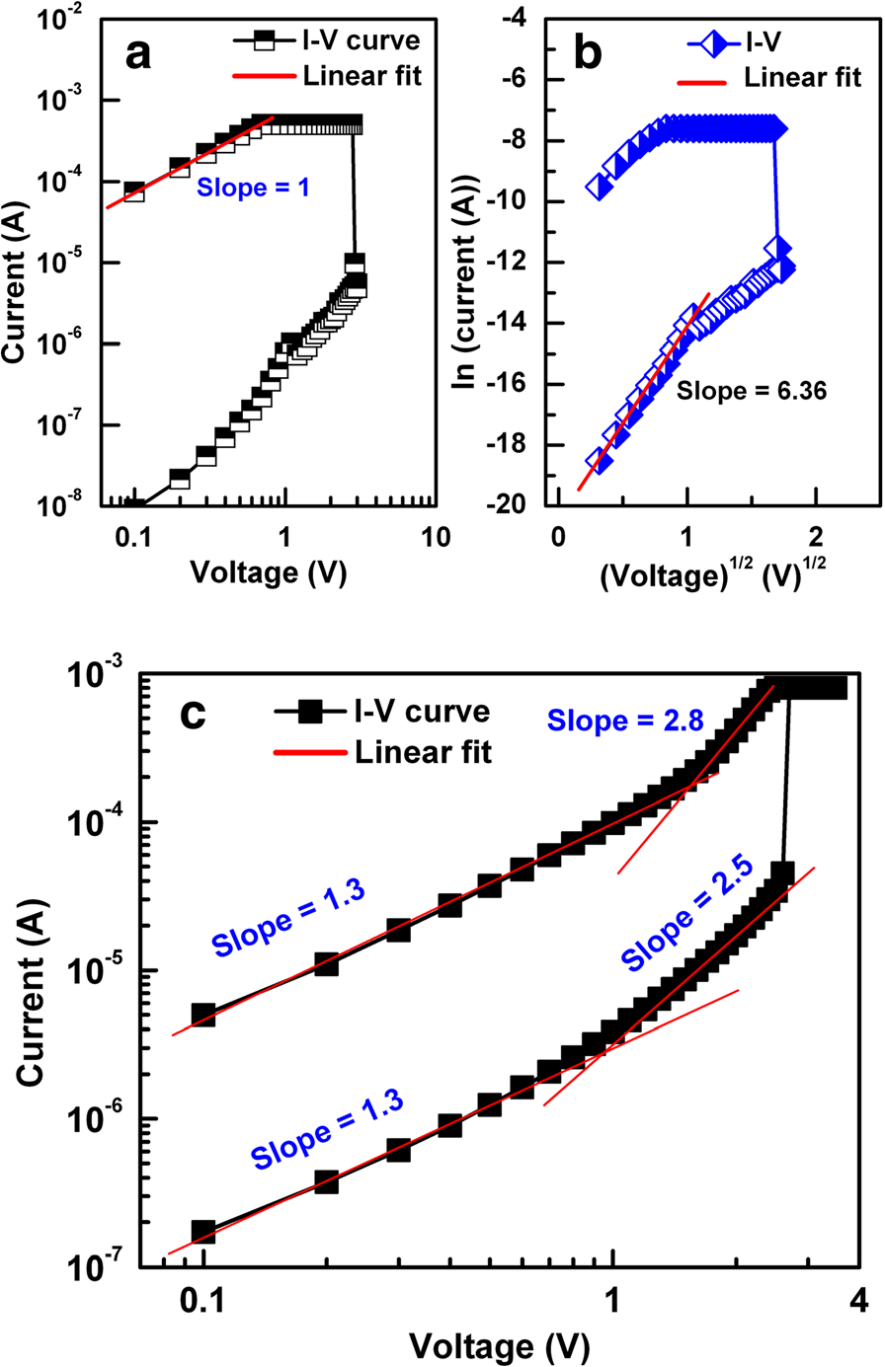
**Fitted *I-V *characteristics of PF and NF devices with IrO**_**x**_**/TaO**_**x**_**/W structure. (a)** LRS of NF devices fitted ohmic behavior. **(b)** HRS for the NF devices were consistent with Schottky behavior. **(c)** Both LRS and HRS of the PF devices show a TC-SCLC transport mechanism.

**Figure 7:**
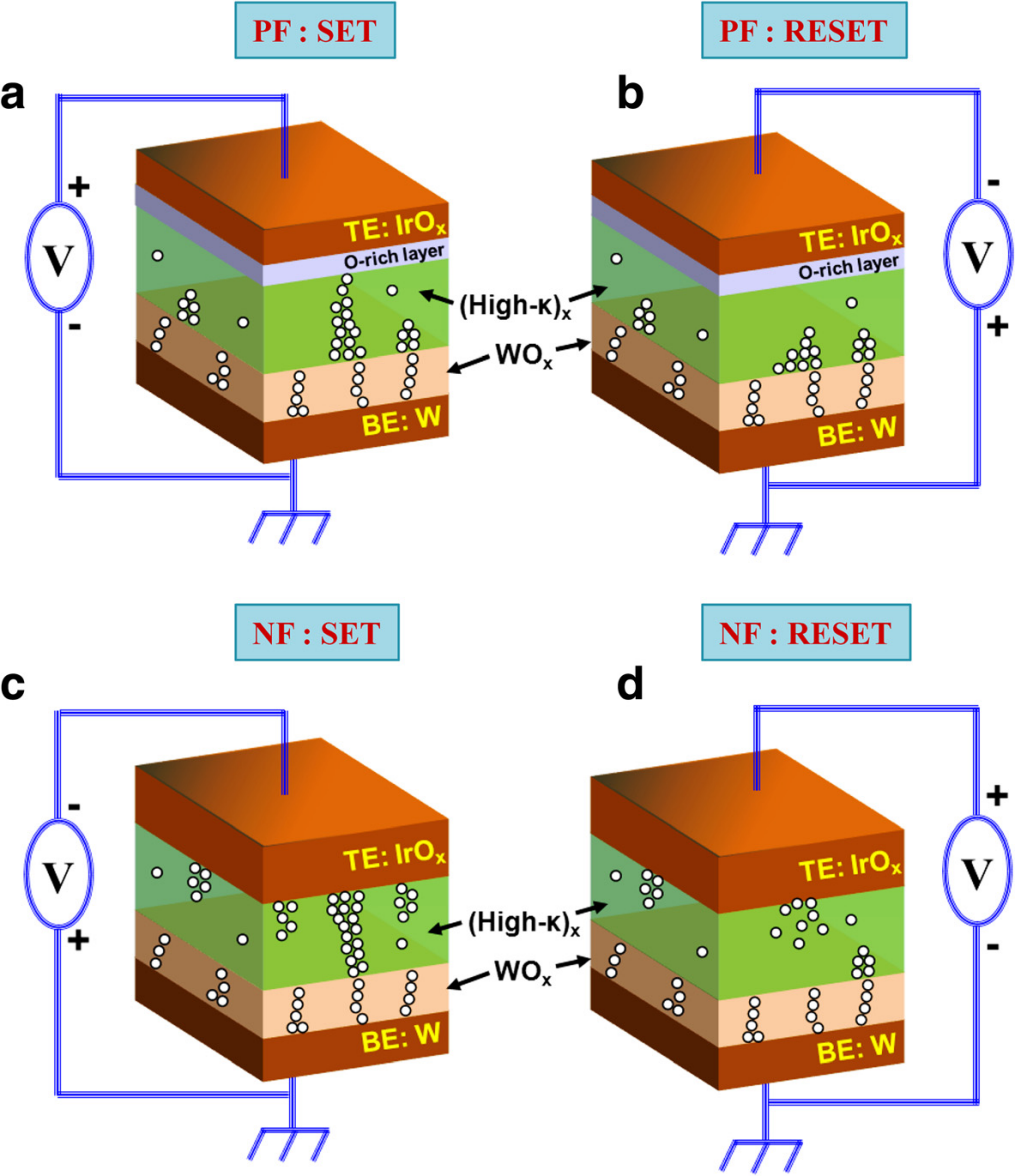
**Resistive switching mechanism of the PF and NF devices.** PF and NF devices under **(a, c)** SET and **(b, d)** RESET operations.

**Figure 8:**
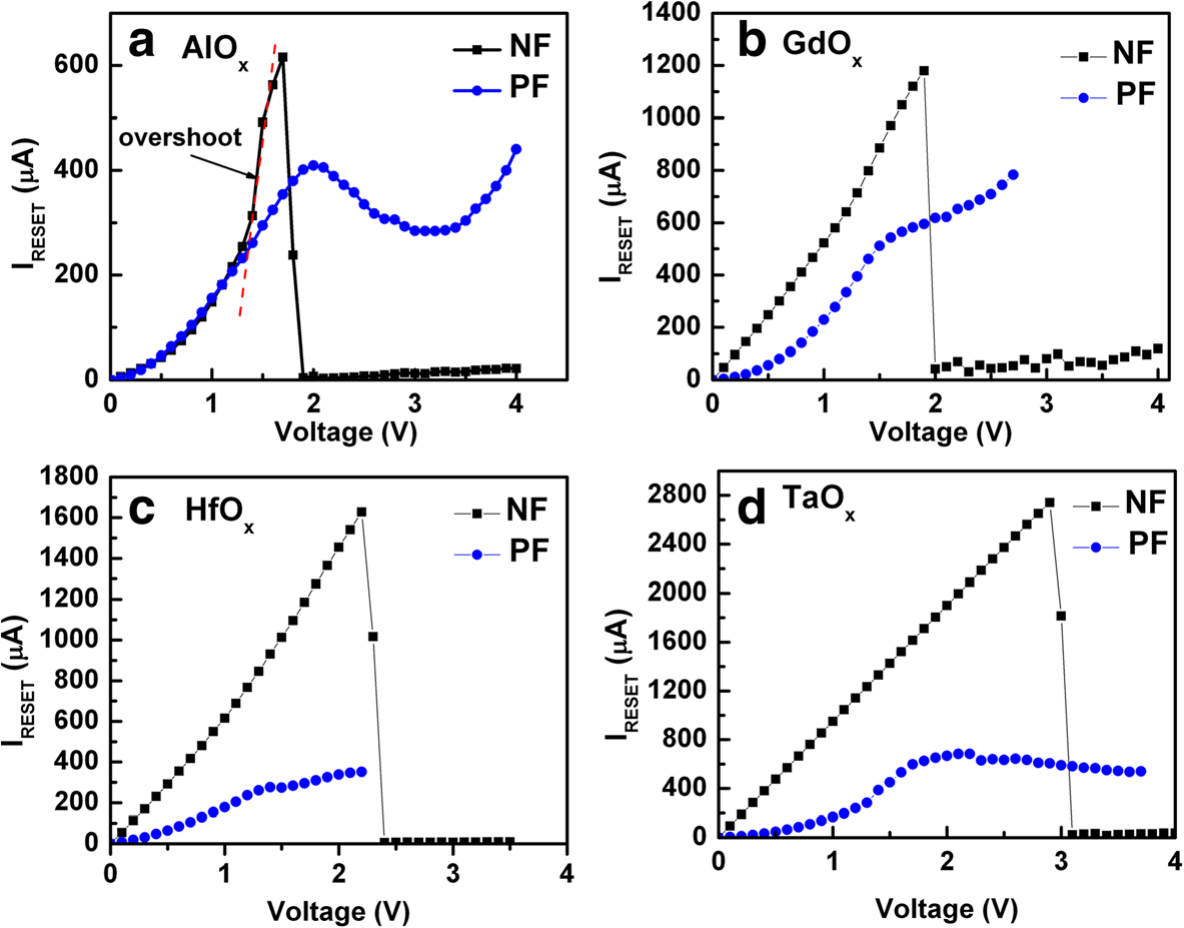
**RESET phenomena for the PF and NF devices.** RESET currents of NF and PF devices containing **(a)** AlO_x_, **(b)** GdO_x_, **(c)** HfO_x_, and **(d)** TaO_x_ switching materials with an IrO_x_/high-κ_x_/W structure.

To determine the current conduction mechanism in the devices, the *I-V* curves of the HRS and LRS of the NF (Figure 6a,b) and PF (Figure 6c) devices with an IrO_x_/TaO_x_/W structure were replotted and fitted linearly. For the NF devices, the LRS was fitted to ohmic conduction with a slope of approximately 1, whereas HRS was consistent with the Schottky emission model. Both LRS and HRS were consistent with a trap-controlled (TC) space charge-limited conduction (SCLC) mechanism following ohmic conduction in the low-voltage region and square law in the high-voltage region for the PF devices. When the positive/negative sweep voltage increases in a pristine device, the metal (M)-O bonds in high-κ oxides AlO_x_, GdO_x_, HfO_x_, and TaO_x_ break and the generated oxygen ions (O^2−^) will drift towards TE or BE according to the direction of the applied field. When a sufficient number of O^2−^ions are generated, the current suddenly increases because of the formation of a conducting filament and the device enters the SET state. In PF devices, the migrated O^2−^form an O-rich layer that is comparatively insulating (i.e., an electrically formed interfacial layer) at the TE/high-κ interface because of the inert nature of the IrO_x_ electrode (which even rejects oxygen) under SET operation (Figure 7a). This interface acts as a series resistance and helps to reduce the overshoot current (Figure 8) as well as increasing the LRS (10 kΩ for PF devices vs. 1 kΩ for NF devices). This is why the PF devices show improved switching properties compared with the NF ones. Under RESET operation of a PF device, O^2−^will be repelled away from the TE and oxidize the oxygen vacancies in the filament, converting the device into a HRS (Figure 7b). Conversely, when O^2−^ions migrate towards the BE in the case of NF devices, they will react with the semi-reactive, partially oxidized W BE to form WO_x_, which can serve as an oxygen reservoir without changing its conductivity. This results in the formation of multiple oxygen filaments (Figure 7c). Under RESET operation of the NF devices, both Joule heating and O^2−^migration from the W BE/high-κ_x_ interface will lead to the oxidation of the conducting filament (Figure 7d). Overshoot RESET current is also observed (Figure 8). The maximum *I*_RESET_ of the devices containing AlO_x_, GdO_x_, HfO_x_, and TaO_x_ switching materials were 616, 1,180, 1,628, and 2,741 μA, respectively, for NF devices, and 409, 543, 276, and 684 μA, respectively, for the PF devices (Figure 8a,b,c,d). The RESET current of NF devices is higher in all cases than the PF devices probably because of higher current overshoot in the NF devices. Current overshoot degrades the switching material because uncontrolled oxygen vacancy filaments form. For the NF devices, the multifilaments can be formed due to oxygen ion migration [39]; however, the filaments are ruptured by thermal effect under RESET operation, i.e., the thermal dissolution of oxygen vacancy filaments may result the uncontrolled filaments to break as well as the SET operation will not be controlled in consequence. The thermal dissolution of conducting filaments under RESET operation on NiO_x_-based resistive switching memories was also reported by Ielmini et al. [25] and Long et al. [40,41]. In contrast, the damage is negligible in the PF devices because of the presence of an electrically formed interfacial layer at the TE/high-κ interface. The filament diameter is readily controlled in the PF devices because of the electrically formed interface. This kind of asymmetric resistive memory stack will help to optimize resistive switching and device performance.

**Figure 9:**
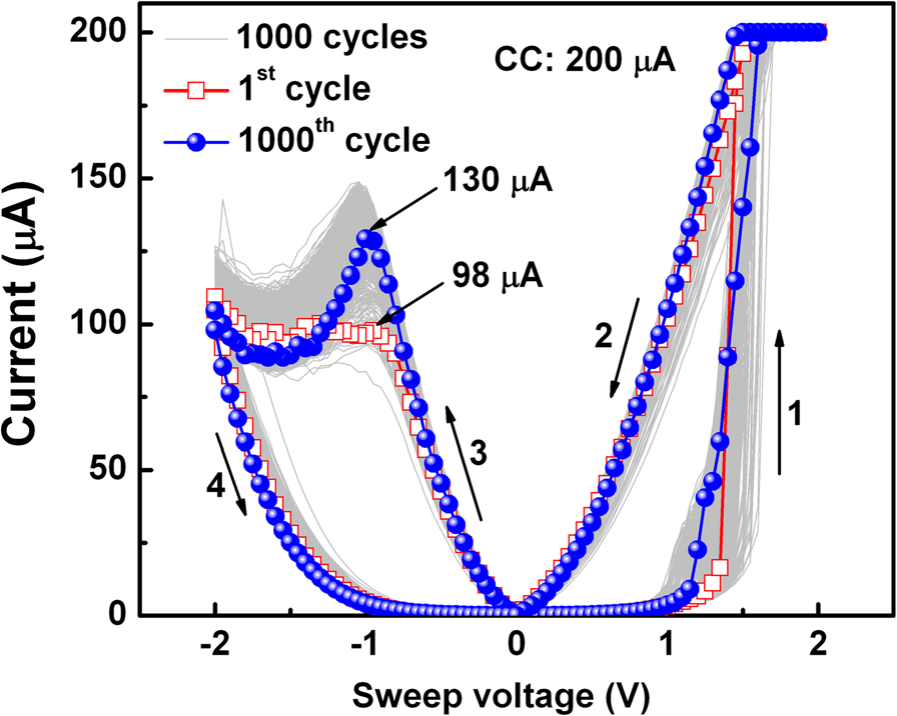
**One thousand consecutive dc switching cycles of IrO**_**x**_**/AlO**_**x**_**/W cross-point memory.** The switching was obtained at a CC of 200 μA and a low operation voltage of ±2 V for the PF device with a size of 4 × 4 μm.

**Figure 10:**
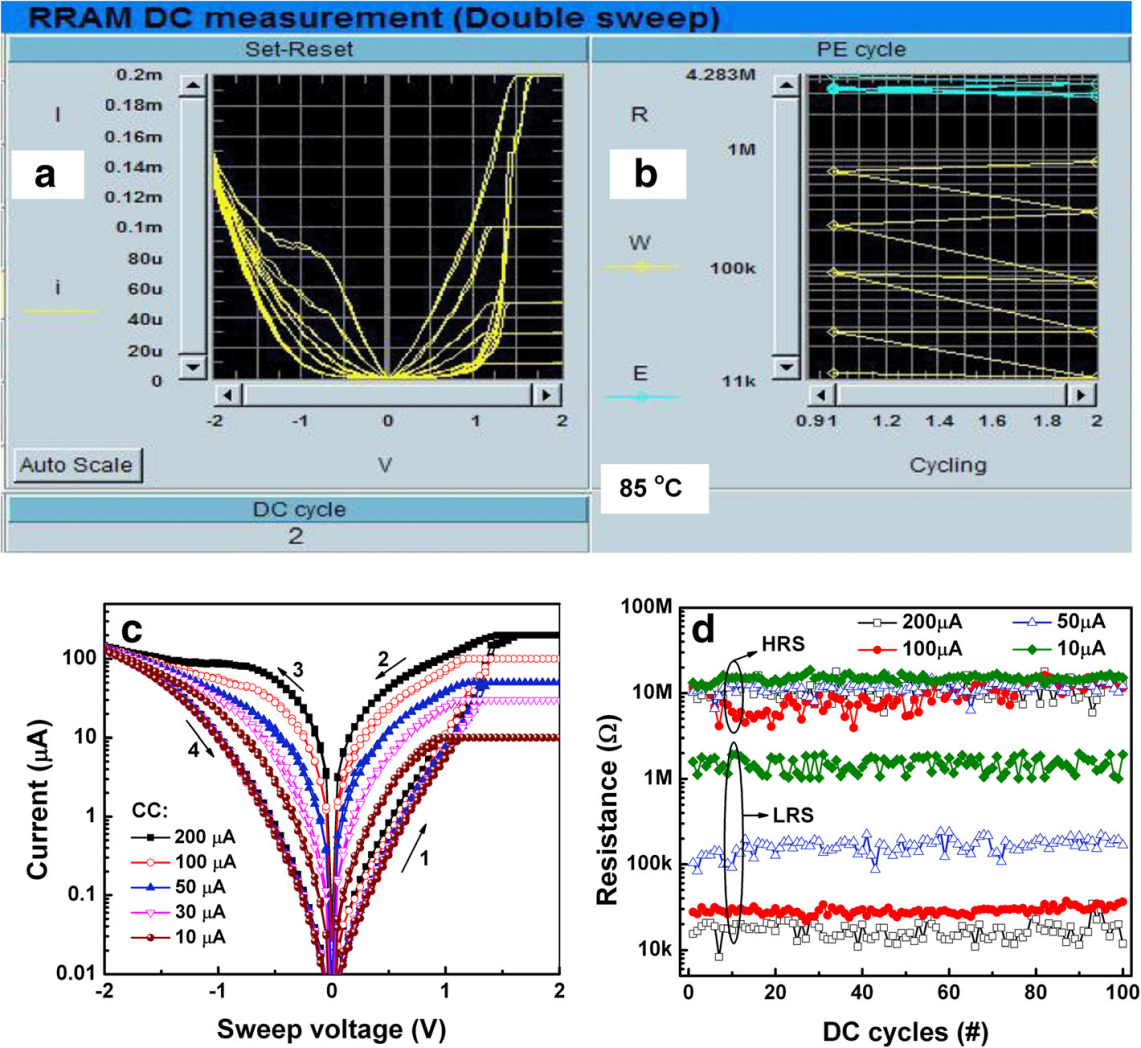
***I-V *switching characteristics and multilevel operation of a cross-point device. (a)** This cross-point device was switchable from CC of 10 to 200 μA at 85°C. Two cycles of each level in linear scale are shown. **(b)** LRS decreases with increasing CC from 10 to 200 μA, whereas HRS remains unchanged. This RRAM device was measured using an interfacing auto program between HP 4156C and a computer. **(c)***I-V* characteristics measured at 85°C replotted in semi-log scale. **(d)** One hundred repeatable switching cycles were observed with CC of 10, 50, 100, and 200 μA.

**Figure 11:**
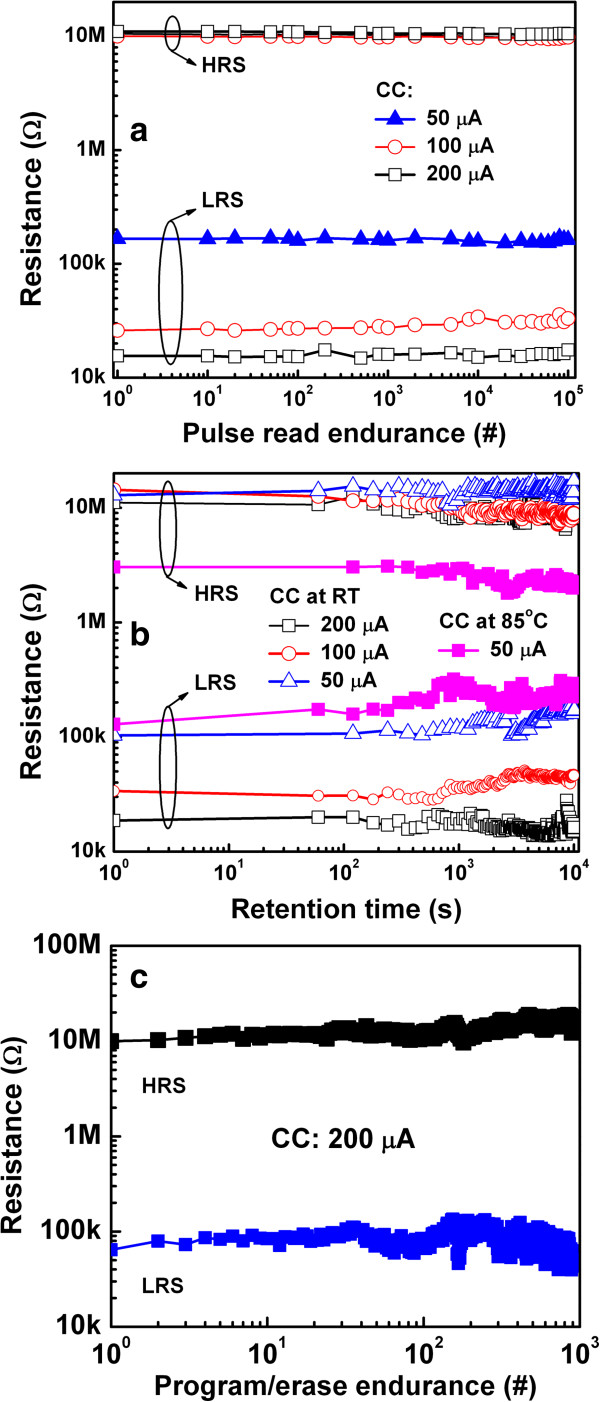
**Stability and data retention of a cross-point device. (a)** Long read pulse endurance of >10^5^ cycles and **(b)** data retention of >10^4^ s are observed with CCs of 50, 100, and 200 μA. Good data retention is also observed at 85°C at a low CC of 50 μA. **(c)** Program/erase endurance of memory device.

High-density memory devices are required for future applications. Resistive memory devices with cross-point architecture show promise to achieve high-density memory. Therefore, we fabricated the resistive memory stacks of IrO_x_/high-κ_x_/W with cross-point structure (S2). Figure 9 shows the typical *I-V* curves of 1,000 consecutive dc switching cycles obtained for an IrO_x_/AlO_x_/W stack. The applied voltage sweep direction is indicated by arrows marked 1 to 4. It is interesting to note that no formation process was required to obtain switching, which is very useful for practical realization of RRAM. Excellent bipolar resistive switching is obtained with a small SET/RESET voltage of approximately ±1.2 V. Furthermore, these results show that a rough surface with nano tips (Figure 4) enhances the electric field on the tips and makes it easier to control the switching cycles. To enhance the resistive switching memory performance, the Cu nanocrystals (NCs) in an Ag/ZrO_2_/Cu-NC/Pt structure was also reported by Liu et al. [42,43]. They mentioned that the electric field could be enhanced and controlled through Cu NC and hence improve the switching characteristics. In our device, a large resistance ratio of >100 with a small operation voltage of ±2 V and CC of 200 μA were obtained for the IrO_x_/AlO_x_/W stack. *I*_RESET_ increased from 98 to 130 μA from 1 to 1,000 cycles, which indicates stronger filament formation after a few switching cycles. A similar increase in RESET current with switching cycle was also reported for a Cu/Ti/TaO_x_/W structure [10]. All cross-point memory devices showed excellent switching with high yields of >95%, which is suitable for nonvolatile memory applications. Both the LRS and HRS were stable during the 1,000 cycles with a narrow distribution of SET/RESET voltages and ratio of LRS to HRS. The underlying switching mechanism was the formation/oxidation of oxygen-vacancy filaments, which was controlled by the electrically formed oxygen-rich layer formed at the TE/AlO_x_ interface under an external field, as for the via-hole devices (S1). The memory devices can be used for multilevel data storage even under harsh conditions (85°C). Figure 10a shows an image of our auto measurement program screen during multilevel capability testing of a device. Linear *I-V* curves at five different levels of LRS are obtained by controlling the CCs from 10 to 200 μA. The corresponding resistances of the LRS read at +0.2 V are approximately 800, 300, 70, 30, and 12 kΩ for CC of 10, 30, 50, 100, and 200 μA, respectively (Figure 10b). Even though this resistive memory device is switchable at a low CC of 10 μA, its *I*_RESET_ is higher, approximately 137 μA (Figure 10c). Figure 10d shows the dc endurance of the multilevel memory of the same device. The HRS remains almost unchanged when CC is varied from 10 to 200 μA. Each LRS level can be switched uniformly for >100 cycles. Furthermore, pulse read endurance and retention tests of the multilevel of memory device were also performed, as shown in Figure 11a,b, respectively. Each level of LRS and HRS were successfully read for more than 10^5^ cycles at a read voltage of 0.2 V without any disturbance for CC of 50 to 200 μA (Figure 11a). The multilevel LRSs are nonvolatile because the retention test shows good stability of these resistance states for >10^4^ s for CC from 50 to 200 μA at room temperature (Figure 11b). Good data retention of >10^4^ s for a CC of 50 μA at 85°C is also observed. The program/erase endurance of approximately 1,000 cycles of the memory device at a current of 200 μA is also shown in Figure 11c. Notwithstanding, we have used a program/erase pulse of 500 μs due to our system limitation. However, the high switching speed (<0.3 ns) of RRAM in HfO_x_ and TaO_x_-based devices were reported by other research groups [44,45]. The robust electrical performance of these essential memory properties makes this device very promising for future high-density nanoscale nonvolatile memory applications.

## Conclusions

Improved resistive switching characteristics independent of switching material are observed for IrO_x_/high-κ_x_/W stacks with a via-hole structure fabricated by positive formation because they contain an electrically formed interfacial layer. High-κ oxides AlO_x_, GdO_x_, HfO_x_, and TaO_x_ were used as switching materials, and similar switching behavior with improved switching uniformity was obtained because overshoot current was minimized in the via-hole structure. AFM images revealed that the BEs of cross-point devices had a higher surface roughness than that of the via-hole devices, which facilitated forming-free switching, improving the switching characteristics. Excellent resistive switching was obtained in Ir/AlO_x_/W cross-point structures using the same PF via-hole design. These devices showed forming-free resistive switching with excellent switching uniformity (>95% yield) over 1,000 dc cycles (approximately 1,000 ac cycles) under low operation voltage/current of ±2 V/200 μA. Multilevel LRSs were obtained by controlling the CCs from 10 to 200 μA with a pulse read endurance of >10^5^ cycles for each level and data retention at room temperature and 85°C under a low CC of 50 μA. This study reveals a route to design nanoscale nonvolatile memory with improved characteristics.

## Competing interests

The authors declare that they have no competing interests.

## Authors’ contributions

AP fabricated and analyzed both the TaO_x_ and HfO_x_ memories and developed the auto measurement program. WB fabricated the AlO_x_-based memory. DJ fabricated the GdO_x_-based memory. This research work was carried out under the instruction of SM. CSL offered the fabrication process. All of the authors revised the manuscript. All authors read and approved the final manuscript.
